# Guidelines for an optimized differential centrifugation of cells

**DOI:** 10.1016/j.bbrep.2023.101585

**Published:** 2023-11-22

**Authors:** Mohammad Sharifian Gh., Fatemeh Norouzi

**Affiliations:** Department of Cell Biology, University of Virginia, Charlottesville, VA, USA

**Keywords:** Centrifugation, Cell, Optimum sedimentation

## Abstract

Literature reviews reveal a significant deficiency in conceptual comprehension concerning centrifugation, a crucial step in both medical and research protocols. The arbitrary fluctuations in centrifugal forces present a potential threat to the reproducibility of results. To address this, we propose concise guidelines that integrate key factors such as temperature, osmolarity, fluid volume, and viscosity. These guidelines aim to enhance comprehension of optimal sedimentation conditions for cell suspensions. Additionally, we introduce a standardized protocol for determining the optimal RCF and centrifugation time. The goal is to maximize sedimentation efficiency while minimizing cell damage, contributing to a universally applicable and reproducible method in centrifugation practices.

## Highlights

• Lack of conceptual understanding of centrifugation endangers result reproducibility in medical and research procedures.
• Temperature, osmolarity, fluid volume, and viscosity play crucial roles in achieving optimal sedimentation of cells.
• An effective protocol for utilizing centrifugal force to attain optimal cell sedimentation is required.

## Graphical abstract

**Figure:**
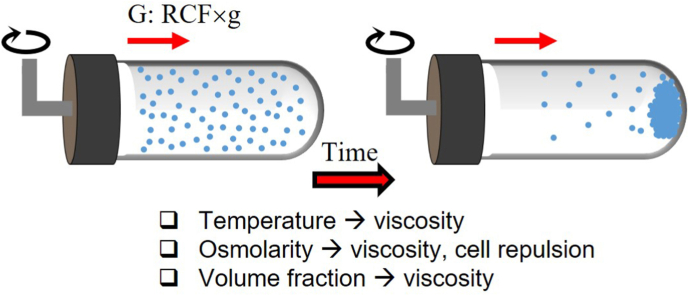

## 1 Introduction

Centrifugation is a frequently utilized technique in medical and research laboratories for isolating distinct cell populations and cell components from their medium, as well as for precipitating cells in bodily fluids or separating fluids with differing densities by means of a centrifugal force greater than that of gravity, i.e., g. Centrifuges can be classified based on their intended usage (differential versus density gradient), rotor design (fixed-angle, swinging-bucket, and right-angle), or rotational speed, including very low (≤4,000×*g*), low (up to 10,000×*g*), high (up to 50,000×*g*), and ultra (≥50,000×*g*) speeds. In ‘differential centrifugation’, dispersions consisting of components with varying densities or sizes are separated based on their sedimentation rates in a density-homogenized suspension. On the other hand, ‘density gradient centrifugation’ separates components into narrow zones that are layered atop each other, depending on their size and mass (rate-zonal) or density (isopycnic). Herein, we focus on differential centrifugation, which is widely employed in separating cells or subcellular fractions in a suspension.

Despite the significant role of differential centrifugation in medical and research procedures, a wide range of centrifugal forces are frequently employed without explicit justification (as outlined in Table 1), thereby posing a risk of potential damage or loss of cells that can compromise the reproducibility of results. For instance, shear forces arising from compaction can affect cell surface-sensitive properties, such as cell deposition on surfaces and cell surface charges [1], and can also lead to a significant reduction in the viability [2] and virulence of bacterial cells [3] at higher forces. The outcome is also influenced by the type of resuspension medium and salt concentration [2]. In addition, high centrifugal force-induced platelet activation has been shown to cause erroneous coagulation test outcomes [4]. Conversely, the use of low centrifugal force (or time) may result in the loss of cells in the supernatant. These effects are compounded during sequential centrifugation steps, particularly when changes are made to the volume of cell suspensions, temperature, or salt concentration. Optimizing the key parameters in cell centrifugation will lead to improved separation efficiency and higher quality of isolated cellular constituents, with significant implications for advancing cell biology and pharmaceutical research.

**Table 1:** **Potential discrepancies between recommended and commonly used values for centrifugal force and sedimentation time in cell suspension:** Various combinations of values of volume, cell numbers, RCF, and sedimentation time, including scenarios where parameters are minimized or maximized, result in a wide range of experimental conditions. As a consequence, the disparities between these conditions can become noticeably accentuated. The data presented herein is indicative of numerous scientific publications and diverse cellular sources, including ATCC, Sigma, and Invitrogen, among others [[5], [6], [7]].

		Eukaryote	Prokaryote	
**Volume (ml)**		5–7	1	50
**Medium of cell suspension**		PBS, cell culture medium, LB broth medium, etc. (with various salt concentrations) [8]		
**# of cells**		(1–100)×10^4^	(1–100)×10^7^	(1–5)×10^10^
**Recommended by vendors**	RCF (×1000)	0.125–0.3	4	4
	Time (min)	3–7	5	15
**Commonly employed by users**	RCF (×1000)	0.8–1	1.5–12	4–6
	Time (min)	5–8	2–10	10–20

Here, we present important factors such as temperature, osmolarity, fluid volume, and viscosity to provide a comprehensive understanding of optimal sedimentation conditions of cell suspensions. Subsequently, we outline a succinct protocol for achieving optimized sedimentation of cell samples in a specific experimental setting.

## 2 Differential centrifugation

**Fig. 1:**
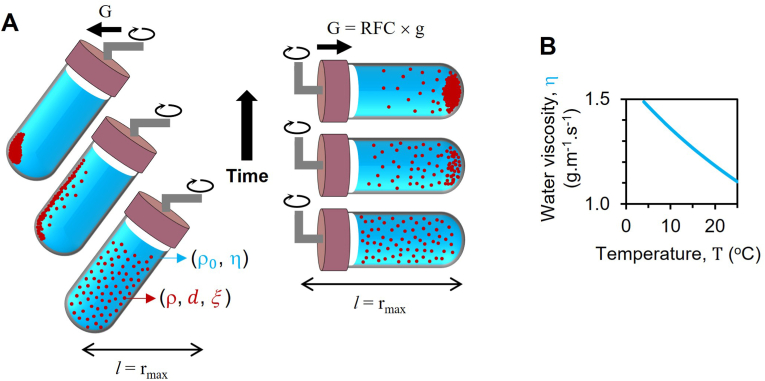
**Experimental principles of cell sedimentation utilizing a centrifugal force.** (**A)** Fixed-angle (left) and swinging-bucket centrifuges (right) are compared, where differential centrifugation separates dispersions of varying densities or sizes in a homogenized suspension based on their sedimentation rates. The centrifugal force, denoted by G, is displayed using arrows. (**B)** Water viscosity at different temperatures is displayed.

In the process of differential centrifugation (Fig. 1A), the duration of sedimentation of a dispersed sample can be determined through the utilization of Eq. (1): [9]

(1) $$t≅6\pi \times \left(\frac{\eta \times l}{d^{2}\times \left(\rho -\rho_{0}\right)\times G}\right)$$

where,

η: viscosity of a suspension ($\text{kg}.m^{-1}.s^{-1}$)
l: pathlength of suspension in a centrifuge tube (m)
d: average diameter of dispersion (e.g., cell) (m)
$\rho_{0}$ and ρ: densities of solvent and dispersion, respectively ($\text{kg}.m^{-3}$)
G: centrifugal force ($m\text{.}s^{-2}$) which is the ‘relative centrifugal force’, RCF (unitless) times g (9.8 $m\text{.}s^{-2}$)

The unit of measure ‘revolutions per minute’ (RPM; $\min^{-1}$) is commonly used in scientific practice, albeit erroneously. A more appropriate metric is RCF, which can be determined using nomograms supplied by centrifuge manufacturers, or by utilizing the following equation: $\text{RCF}=\left(1.118\times 10^{-3}\right)\times r\times {\text{RPM}}^{2}$, where ‘r’ denotes the distance from the central axis of the centrifuge in meters (Fig. 1A).

**Fig. 2:**
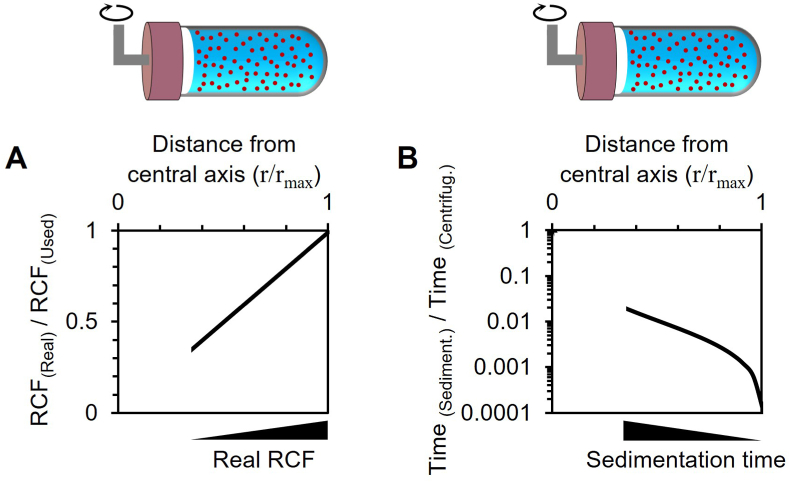
Regional RCF (**A**) and sedimentation time (**B**) at varying distances from the rotation axis.

It is important to note that Eq. (1) accounts for the maximum value of r (i.e., l = $r_{\max}$), as illustrated in Fig. 2. However, particles located closer to the central axis (i.e., those with smaller values of l) experience reduced centrifugal force, and therefore require a longer time to sediment. And, particles in proximity to the bottom of the centrifuge tube (i.e., those with larger values of l) experience greater centrifugal force during sedimentation, causing them to be compressed by the tube wall before complete sedimentation is achieved. This phenomenon becomes particularly relevant in experiments involving multiple distinct populations [10]. Despite this issue, density gradient centrifugation enables the separation of components into narrow zones based on their size, mass, or density, which stack on top of each other. In the present work, we focus on a simpler scenario in which the aforementioned complication can be largely disregarded, and Eq. (1) can be utilized in a simplified format during differential centrifugation.

## 3 Impactful parameters in cell centrifugation

The terms relating to solvent and dispersion within Eq. (1) may be categorized as either independent (i.e., $\rho_{0}$, ρ and d) or dependent (i.e., η) upon experimental variables. Factors such as temperature, salt concentration, and the volume fraction of the dispersed phase (e.g., cells) can exert an influence upon η, as well as two additional dependent variables - namely, the correction coefficient (β) for η, and the zeta potential (ξ) of the cellular surface. The precise effects of such variables are detailed as follows:

### 3.1 Temperature

Although it is advisable to conduct experiments at lower temperatures (e.g., 4°C) in order to minimize temperature-induced biological effects, such as protein degradation, it is worth noting that alterations in temperature can also have an impact on the centrifugation process, owing to the temperature-dependence of η [11].

(2) $$\eta =\frac{hN_{A}}{\bar{V}}e^{\left(\frac{\Delta G}{\text{RT}}\right)}$$

where,

h: Planck's constant (6.626 × 10^−34^ J.s)
$N_{A}$: Avogadro's number (6.022 × 10^23^ mol^−1^)
$\bar{V}$: average molar volume (18 × 10^−6^ m^3^^.^mol^−1^ for water)
ΔG: Gibbs free energy of activation for viscous flow
R: gas constant (8.314 J K^−1^^.^mol^−1^)

As indicated in Fig. 1B, the information illustrates that with the viscosity of water at 4°C and 25°C being 1.49 g.m^−1^^.^s^−1^ and 1.11 g.m^−1^^.^s^−1^, respectively, and holding all other factors in Eq. (1) steady, a shift in temperature from 4°C to 25°C can result in a 25% alteration in the optimal sedimentation time or RCF required for the sedimentation of a dispersed substance.

### 3.2 Osmolarity

Biological media and solvents are composed of various concentrations of ions, such as Na^+^, K^+^, Ca^2+^, Mg^2+^, NH_4_^+^, Cl^−^, SO_4_^2−^, PO_4_^3−^ etc., that serve to mitigate the effects of osmotic shock. In the context of serial centrifugation, it is conceivable that fluctuations in the concentrations of cations and anions may occur as a result of the use of differing media or buffers. These changes have the potential to influence centrifugation yield via two distinct pathways:

#### 3.2.1 Viscosity

The interplay between ions and water molecules results in alterations to the structure of water, thereby affecting its viscosity. These ions can be classified as either kosmotropes, which are structure makers, or chaotropes, which are structure breakers [12]. In the realm of low electrolyte concentrations, where the concentration does not exceed 100 mM, the Jones-Dole equation is the prevailing equation utilized to establish a correlation between the concentration dependence of an electrolyte solution's viscosity: [13]

(3) $$\frac{\eta }{\eta_{0}}=1+A\sqrt{C_{\text{salt}}}+BC_{\text{salt}}$$

In Eq. (3), the symbols η and $\eta_{0}$ represent the dynamic viscosities of the solution and the pure solvent, respectively, while $C_{\text{salt}}$ indicates the molar concentration of the electrolyte. The coefficient ‘A’ is linked to the conductance of ions under conditions of infinite dilution, in accordance with the Debye-Huckel theory [14]. Conversely, the coefficient ‘B’ is determined through experimental data fitting and characterizes the extent of water structuring. For kosmotropic ions, this coefficient possesses a positive value, while chaotropic ions are associated with a negative value [15,16].

The viscosity of electrolyte solutions is known to exhibit anomalous concentration dependence, particularly at higher ionic concentrations [12]. Notably, for certain electrolytes such as NaCl(aq) or LiCl(aq), the viscosity (η) increases monotonically with the concentration of salt ($C_{\text{salt}}$). In contrast, for other electrolytes, such as KCl(aq) or KBr(aq), the viscosity initially increases up to a maximum value (at approximately 50–100 mM), then decreases to a minimum value (at approximately 500–750 mM), and finally increases monotonically at higher concentrations [17,18]. Esteves et al. have proposed a more comprehensive model for computing the viscosity of binary strong electrolyte solutions [19].

In summary, altering the type and/or molar concentration of ions can affect the η value of a suspension, which, in turn, impacts the optimal sedimentation time. Typically, variations in the viscosity of water by 1–5% are observed when the electrolyte concentration of the suspension changes within the range of 100–500 mM [17,18].

#### 3.2.2 Cell-cell repulsion

The surface charge density of prokaryotic [20] and eukaryotic cells [21] is contingent upon the physiological state and molecular composition of the cell membrane. Notably, the lipopolysaccharide-coated surface of Gram-negative bacteria exhibits a negative charge density (6.6 ± 1.3 nm^−2^ for *E. coli*) that is up to seven times greater than that of the protein surface layer of Gram-positive cells at physiological pH (1.0 ± 0.2 nm^−2^ for *L. rhamnosus*) [22]. To determine the cell surface charge, zeta-potential (ξ) is typically utilized, which measures the electrical potential of the interfacial region between the cell surface and the local environment. The ξ values usually range from −50 to −20 mV for different cell types [22,23] or extracellular vesicles [24] under varying salt and detergent concentrations.

The ξ term serves as an indicator of the repulsive interaction between cells, whereby at zero potential, the conditions for cellular aggregation are maximized. The repulsive force between surface-charged dispersions is roughly proportional to the square of the ξ value (according to Coulomb's law) [25]. The ionic strength of a suspension has a significant impact on the ξ value, wherein the presence of ions results in a less negative ξ value, particularly for ions with higher valency, such as Ca^2+^ [1,[22], [23], [24],^26^]

To summarize, a higher concentration of salts leads to a more rapid sedimentation of cells. Altering the salinity during serial centrifugation steps may result in the loss of cells in the supernatant or possibly damage the cells due to the application of excessive centrifugal force.

### 3.3 Volume fraction of cells

In order to account for the influence of the volume fraction of the dispersed phase (φ, unitless) on the viscosity (η) as described in Box 1, it is necessary to apply a correction coefficient for viscosity, β (unitless), to Eq. (1) [27]. As φ increases, particularly for lower $\varphi^{*}$ values, the β value increases. For instance, when the volume fraction of a suspension of eukaryotic cells changes from 10^3^ cells.ml^−1^ (φ ≈ 6.5 × 10^−5^) to 10^4^ cells.ml^−1^ (φ ≈ 6.5 × 10^−4^), the optimal sedimentation time or RCF will increase by 22.5 % (if $\varphi^{*}$ = 0.01) or by 2.3 % (if $\varphi^{*}$ = 0.1) (see Box 1). Therefore, employing constant values for time and RCF during consecutive centrifugation steps will result in either loss of cells (at higher φ values) or excessive centrifugal force on the cells (at lower φ values), which is particularly pronounced for cells with lower $\varphi^{*}$ values.

Box 1 The βdependence onφandφ*.

The β coefficient (unitless) incorporates the influence of the volume fraction (φ) on the intrinsic viscosity ([η]), where [η] (unitless) is defined as the limit of (η - $\eta_{0}$)/($\varphi \eta_{0}$) as φ approaches zero. Here, $\eta_{0}$ and η represent the viscosities of the pure solvent and the suspension, respectively: [27,28]

(8) $$\beta ≅1+\left[\eta \right]\varphi +k_{H}\varphi^{2}$$

Theoretical computations of the Huggins coefficient, denoted as $k_{H}$ (unitless), anticipate a range of values spanning from 5.0 to 5.2 for repulsive spherical particles. Nonetheless, the effects of Brownian motion on diminutive dispersions escalate the predicted values to 6.0 to 6.2 [28]. The coefficient β is reliant not only on the concentration of the dispersed phase but also on the ratio of the dispersed phase viscosity to that of the pure solvent. As a result, β diverges as the volume fraction of dispersed phase (denoted as φ) approaches the critical value $\varphi^{*}$ (described by Eq. (9)). $\varphi^{*}$ is interpreted as the volume fraction at which the dispersion undergoes a transition from a fluid to a non-fluid state. The precise value of $\varphi^{*}$ depends on the particle shape, the propensity for aggregation, and the rate of shear applied to the suspension [27].

(9) $$\beta ≅{\left(1-\frac{\varphi }{\varphi^{*}}\right)}^{-2}$$

A proposition is put forth for a crossover equation that smoothly transitions between the semi-dilute and concentrated regimes of a suspension, incorporating both Eq. (8) and Eq. (9). This proposal is put forward with the aim of providing a more systematic and rigorous framework for analyzing the behavior of suspensions in these regimes: [27]

(10) $$\beta ≅\frac{1+\left[\eta \right]\left(\varphi -\frac{{2\varphi }^{2}}{\varphi^{*}}\right)+\frac{\varphi }{\varphi^{*}}\;\left(\frac{\varphi }{\varphi^{*}}-2\right)+k_{H}\varphi^{2}}{{\left(1-\frac{\varphi }{\varphi^{*}}\right)}^{2}}$$

The term β can be expressed as a function dependent solely on the variables φ and $\varphi^{*}$, given that the [η] is proportional to $\varphi^{*}$. As an instance, [η] can be approximated as 1.7/ $\varphi^{*}$ [29].

Alt-text: Box 1

## 4 A standardized protocol for optimizing cellular sedimentation via centrifugation

The absence of a standardized protocol that comprehensively considers all crucial parameters involved in cell centrifugation, coupled with the prevalent practice of disseminating suboptimal protocols that are not tailored to specific cell samples, underscores the imperative for a universal protocol. Accordingly, we propose a succinct yet universally applicable protocol to achieve optimal sedimentation of cell samples within a specified experimental framework. Our proposed protocol to determine optimal centrifugation time and RCF values, involves three steps, as follow:

Step (1): Assessing the potential impact of the centrifugation on the overall experimental procedure

**Fig. 3:**
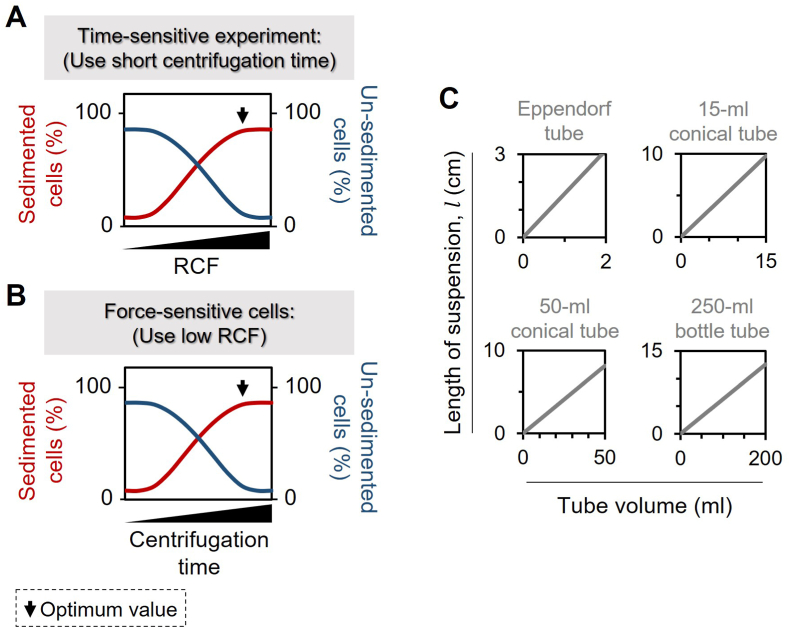
A broadly applicable guideline is provided for establishing the ideal RCF in time-sensitive experiments (**A**) or the optimal sedimentation time for cells sensitive to force (**B**). This guideline maintains constant values for solvent and dispersion parameters, including temperature, tube length, salt concentration, and volume fraction of cells. (**C**) The magnitude of the l value will vary based on the volume of the suspension being subjected to centrifugation, which, in turn, depends on the shape of the centrifugation tube (i.e., Eppendorf, conical, or bottle tubes).

As depicted in Fig. 3, the suggested guideline proves beneficial in scenarios involving (i) time-sensitive experiments, requiring a swift completion of the sedimentation step, or (ii) force-sensitive cells where there's a concern that higher centrifugal forces might negatively impact cellular physiology. For situation (i), one should experimentally establish the RCF value for a brief, fixed centrifugation time (e.g., 1 min) that yields the maximum cell pellet (see Fig. 3A). Conversely, in case (ii), it becomes crucial to ascertain the time value for a fixed, low RCF (e.g., 100), resulting in the maximum cell pellet (Fig. 3B). Note that several other experimental variables, including the solvent's viscosity (η) and density ($\rho_{0}$), cell size (d), cell density (ρ), and volume fraction (φ and $\varphi^{*}$), as well as the suspension's length (l), temperature, and salt concentration ($C_{\text{salt}}$), are all maintained constant.

Step (2): Experimentally identifying the optimal values for RCF and centrifugation time

**Fig. 4:**
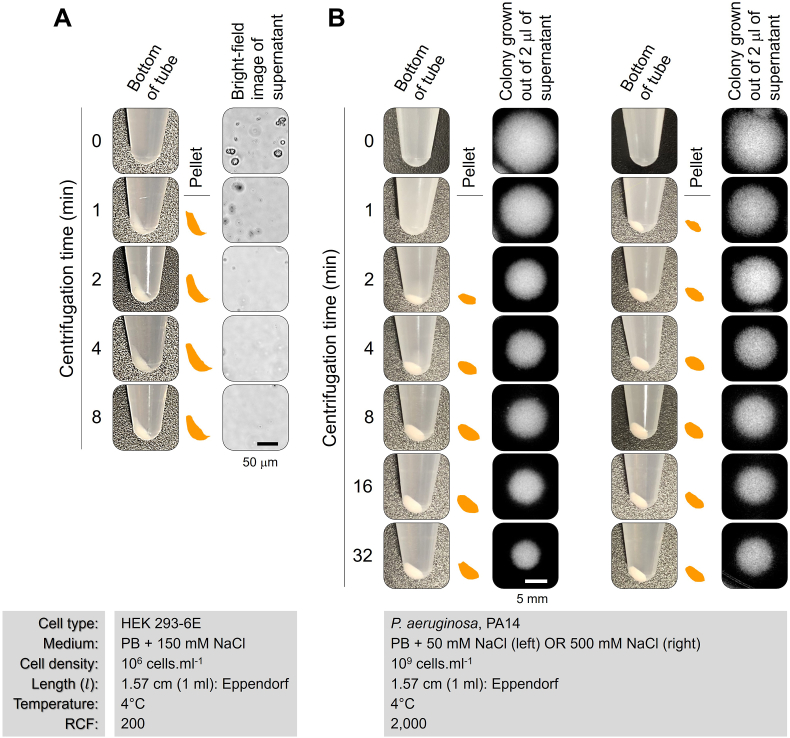
**A straightforward protocol for achieving optimal sedimentation of cells via centrifugal force.** Experimental illustration demonstrating the centrifugation process for a type of (**A**) eukaryotic cells (e.g., Human Embryonic Kidney, HEK cell) and (**B**) bacterial cells (e.g., *Pseudomonas aeruginosa*). Schematic representations highlight sedimented cells at the bottom of Eppendorf tubes. The presence of un-sedimented cells grown on LB agar plates (part (**B**)), even after 32 min of centrifugation, underscores the challenge of achieving 100 % sedimentation for small-sized bacterial cells.

After determining the optimal approach between utilizing constant centrifugation time to establish the optimal RCF or determining the optimal centrifugation time by maintaining a constant RCF (Step 1), it is essential to conduct a sample experiment. Fig. 4 presents a straightforward protocol that is applicable to various cell types under different conditions.

Fig. 4A presents an example of employing differential centrifugation to separate 1 ml of 10^6^ cells/ml of a force-sensitive eukaryotic cell in an Eppendorf tube. The process involved applying a low RCF of 200 at 4°C, maintaining physiological salt concentration. At various time points, 5 μl of the supernatant was scrutinized using a bright-field transmission microscope. Schematic representations were utilized to enhance the visibility of the pellets, which represent the sedimented cells. These findings indicate that a centrifugation time of 2 min is sufficient to precipitate the majority of cells. Note that this is significantly lower than the times (and RCF) typically employed by users, as documented in Table 1.

In Fig. 4B, a similar protocol was followed using a bacterial cell type with a cell density of 10^9^ cells/ml. The centrifugation involved an RCF of 2,000 and was conducted under two different osmolarities. The findings indicated that pellets were distinctly visible following 1 min of centrifugation at a salt concentration of 500 mM, whereas at a lower salinity of 50 mM, pellets became apparent only after 2 min (i.e., as expected due to the impact of osmolarity on cell-cell repulsion). 2 μl of the supernatants at each time point of centrifugation were then transferred to an LB agar plate for overnight growth. As shown, even after 32 min of centrifugation, there were still un-sedimented cells in the supernatant that grew on the plate. This suggests that achieving 100% sedimentation is challenging for small-sized cells like bacteria, even with prolonged centrifugation. Consequently, it is highly recommended to avoid extended periods (or high RCF) in such cases.

Step (3): To obtain the optimal RCF or centrifugation time values under diverse conditions

Upon conducting experimental analysis of a particular cell sample, the optimal values of RCF and sedimentation time (RCF_1_ and t_1_) can be determined. Subsequently, utilizing Eq. (4) or (5) enables the acquisition of optimum RCF or time values across varying parameters where the cell type and solvent remain constant, but the temperature, salt concentration, volume fraction, or suspension's length may vary. Both equations are derived from Eq. (1) under two distinct conditions represented by subscripts 1 and 2. Note that terms such as 6π, $d^{2}$, and ($\rho -\rho_{0})$ in Eq. (1) are eliminated as their values remain unchanged under both conditions.

(4) $$t_{2}≅\left\{\frac{\beta_{2}}{\beta_{1}}\times \frac{l_{2}}{l_{1}}\times \frac{\eta_{2\left(T,C_{\text{salt}}\right)}}{\eta_{1\left(T,C_{\text{salt}}\right)}}\times {\left(\frac{\xi_{1}}{\xi_{2}}\right)}^{2}\times \frac{{\text{RCF}}_{1}}{{\text{RCF}}_{2}}\right\}\times t_{1}$$

(5) $${\text{RCF}}_{2}≅\left\{\frac{\beta_{2}}{\beta_{1}}\times \frac{l_{2}}{l_{1}}\times \frac{\eta_{2\left(T,C_{\text{salt}}\right)}}{\eta_{1\left(T,C_{\text{salt}}\right)}}\times {\left(\frac{\xi_{1}}{\xi_{2}}\right)}^{2}\times \frac{t_{1}}{t_{2}}\right\}\times {\text{RCF}}_{1}$$

It is worth noting that the l value varies differently with the volume of a suspension, depending on the centrifuge tube type (see Fig. 3C).

As already mentioned, altering influential parameters such as temperature, salinity, volume fraction of cells, or even the centrifugation tube can have a substantial impact on the optimized values of RCF and sedimentation time. It is important to note that determining the exact influence of those variables on viscosity of cell suspensions, zeta-potential of cells, and β parameter can be challenging and time-consuming. Consequently, it is strongly advised to maintain a consistent set of values for these parameters throughout a serial centrifugation process. By doing so, the equations (4), (5) can be simplified to equations (6), (7) as follows:

(6) $$t_{2}≅\frac{l_{2}}{l_{1}}\times \frac{{\text{RCF}}_{1}}{{\text{RCF}}_{2}}\times t_{1}$$

(7) $${\text{RCF}}_{2}≅\frac{l_{2}}{l_{1}}\times \frac{t_{1}}{t_{2}}\times {\text{RCF}}_{1}$$

As an illustration, in a time-sensitive experiment, if an optimized certification time of 1 min and an RCF of 2,500 are determined for a specific cell sample, extending the length of the cell suspension by two times and applying a sedimentation time of 30 s would necessitate an RCF of 10,000, assuming that all other parameters remain unaltered (refer to Eq. (7)). Alternatively, in the context of optimizing certification time, if a specific force-sensitive cell sample exhibits an ideal configuration when subjected to a 5-min centrifugation with an RCF of 300, by increasing the duration of the cell suspension twofold and employing an RCF of 200, the optimal sedimentation time extends to 15 min, assuming all other parameters remain unchanged (refer to Eq. (6)).

## 5 Conclusions

A diverse range of centrifugal forces are often employed without explicit justification (Table 1), putting cells at risk of potential damage or loss. Shear forces, which are triggered by compaction, can impact cell surface-sensitive properties. Higher forces can also lead to significant reductions in viability of cells. The outcome is also affected by the type of resuspension medium and salt concentration. Conversely, utilizing low RCF (or time) may result in cell loss in the supernatant. These effects are amplified during serial centrifugation steps, particularly when changes are made to the volume of cell suspensions, temperature, or salt concentration. For example, if one were to switch from utilizing 5 ml, 8°C, and higher salt concentrations (e.g., ξ ≈ −50 mV) to 10 ml, 4°C, and lower salt concentrations (e.g., ξ ≈ −35 mV), the optimal sedimentation time or RCF could differ by up to 400% (according to Eq. (4) or (5)). Therefore, utilizing an unoptimized centrifugation method has the potential to generate non-reproducible experimental results, a crucial aspect that is frequently overlooked by users. Optimizing the influential parameters in cell centrifugation will enhance separation efficacy and improve the quality of isolated cellular constituents, with significant implications for advancing cell biology and pharmaceutical research.

## Declaration of competing interest

The authors declare that they have no known competing financial interests or personal relationships that could have appeared to influence the work reported in this paper.
